# Feasibility of Outpatient Transradial Prostatic Artery Embolization and Safety of a Shortened Deflation Protocol for Hemostasis

**DOI:** 10.3390/jpm12071138

**Published:** 2022-07-14

**Authors:** Gregory Amouyal, Louis Tournier, Constance de Margerie-Mellon, Damien Bouda, Atanas Pachev, Jessica Assouline, Cédric de Bazelaire, Florent Marques, Solenne Le Strat, François Desgrandchamps, Eric De Kerviler

**Affiliations:** 1 Hôpital Privé Geoffroy Saint-Hilaire—Ramsay Santé, 75005 Paris, France; florent.marques@gmail.com (F.M.); docteurlestrat@gmail.com (S.L.S.); 2 Radiology Department, Hôpital Saint-Louis, 75010 Paris, France; ltourn22@gmail.com (L.T.); constance.de-margerie@aphp.fr (C.d.M.-M.); damien.bouda@aphp.fr (D.B.); atanas.pachev@aphp.fr (A.P.); jessica.assouline@aphp.fr (J.A.); cedric.de-bazelaire@aphp.fr (C.d.B.); eric.de-kerviler@aphp.fr (E.D.K.); 3 Faculté de Médecine, Université Paris cité, 75006 Paris, France; francois.desgrandchamps@aphp.fr; 4 Urology Department, Hôpital Saint-Louis, 75010 Paris, France; 5 SRHI/CEA—Institut de Recherche Clinique Saint-Louis, Hôpital Saint-Louis, 75010 Paris, France

**Keywords:** prostatic hyperplasia, embolization, therapeutic, endovascular procedure, radiology, interventional, prostate

## Abstract

Background: to evaluate the safety and feasibility of a shorter time to hemostasis applied to outpatient transradial (TR) Prostatic Artery Embolization (PAE). Methods: a retrospective bi-institutional study was conducted between July 2018 and April 2022 on 300 patients treated by outpatient TR PAE. Indications included lower urinary tract symptoms, acute urinary retention, and hematuria. Mean patient height was 176 ± 6.3 (158–192) cm. The primary endpoint was safety of a 45 min deflation protocol for hemostasis. The secondary endpoint was the feasibility of PAE using TR access. Results: technical success was 98.7% (296/300). There was one failure due to patient height. Mean DAP/fluoroscopy times were 16,225 ± 12,126.3 (2959–81,608) μGy·m^2^/35 ± 14.7 (11–97) min, and mean time to discharge was 80 ± 6 (75–90) min. All access site and embolization-related adverse events were minor. Mild hematoma occurred in 10% (30/300), radial artery occlusion (RAO) in 10/300 (3.3%) cases, and history of smoking was a predictor for RAO. There was no major event. Conclusion: the safety of TR PAE using a 45 min time to hemostasis was confirmed, and TR PAE is feasible in most cases. Radial artery occlusion was still observed and may be favored by smoking.

## 1. Introduction

Prostatic Artery Embolization (PAE) has been proposed for several years as an alternative treatment to surgery for symptomatic Benign Prostatic Hyperplasia (BPH) [1,2]. This endovascular intervention performed by interventional radiologists (IR) has shown safety, efficacy, and comparable outcomes to surgical results [3,4,5,6].

PAE was routinely performed during a short hospital stay using transfemoral access (TFA). However, ambulatory PAE is spreading in most IR institutions, as immediate post-operative symptoms are mild and well tolerated. A few reports on the use of transradial access (TRA) in IR procedures showed safety and multiple benefits for the patient [7,8], such as gains in per- and post-procedural comfort (less discomfort during local anesthesia delivery; possibility for elevation of the legs during the procedure, to relieve back pain; possibility for immediate resumption of standing position and ambulation) [9], decreased rate of hemorrhagic adverse events compared to TFA [10], possibility to maintain antiaggregant or anticoagulant medication, shorter time to hemostasis, and faster discharge during ambulatory stays [8].

TRA for PAE has been described with promising initial experiences in terms of feasibility and safety [9,11]. Hemostasis is obtained using a compressive band, with a step-by-step deflation protocol, the duration of which is not yet standardized in IR procedures. As the main challenge is avoiding subsequent post-compression radial artery occlusion (RAO), reflections have been raised on how to reduce the incidence of this complication, among them being the duration of compression. 

In this study, an assessment of the feasibility and safety was conducted on a cohort of patients who benefited from outpatient Transradial (TR) PAE, using a shortened deflation protocol, with the objectives to lower the incidence of RAO and to shorten the patients’ hospital stay.

## 2. Materials and Methods

This retrospective, bi-institutional study was conducted on 300 male patients, mean age 68 ± 9.7 (47–102) years, who underwent TR PAE between July 2018 and April 2022. This TRA cohort belonged to a population of 311 consecutive patients treated with PAE, using either TRA or Transfemoral access (TFA), all performed as an outpatient procedure. 

Indications for PAE included symptomatic BPH with moderate-to-severe lower urinary tract symptoms and failure of medical treatment, acute urinary retention or macroscopic hematuria due to BPH, and were validated in clinic by a urologist and an IR. Pre-procedural patient assessment was performed as previously described [12].

Ambulatory PAE was performed under local anesthesia (center 1) or anesthesia and neurolept analgesia (center 2) using a subcutaneous peri arterial 4 mL injection of licodaïne mixed with 1 mg of isosorbide dinitrate. 

All patients were treated with the intent to use TRA to increase patient comfort and shorten ambulatory stay. Choice for TFA was made only in cases where TRA faced a risk of failure or morbidity, such as excessive height (>195 cm); mental condition unfit for TRA patient installation, such as agitation or dementia; advanced atherosclerosis (defined by a combination of at least 3 factor risks among diabetes, arterial hypertension, smoking, and dyslipidemia in addition to a history of cardiovascular acute event); or obstacles for catheterization in the thoracic or abdominal arterial territory. 

In case of TRA, antiaggregant, Direct Oral Anticoagulant (DOA), or Vitamin K Agonist (VKA) medications were not discontinued. When TFA was used, aspirin was maintained; clopidogrel, ticagrelor, or DOA medications were discontinued at least 5 days prior to embolization; and VKAs were transitorily replaced by heparin.

Oxymetric Barbeau test was performed to rule out contraindication for radial puncture, followed by pre-operative left radial artery Doppler Ultrasound (DUS): caliber of the radial artery at puncture site, radial artery patency (RAP), and presence of radial loop were monitored. 

Baseline characteristics of the population are presented in Table 1. At time to procedure, 26/300 patients (8.7%) were under anticoagulant medication and 27/300 (9%) under antiaggregant medication. Mean radial artery diameter at puncture site was 2.5 mm ± 0.3 (1.7–3.6), and mean patient height was 176 ± 6.3 (158–192) cm. 

TRA was always performed on the left side, as previously described [9,11], using a dedicated 5-Fr sheath for radial puncture (Merit medical, Salt Lake City, UT, USA), composed of a 21-G needle and a 0.018-inch guide wire. In rare situations of a radial artery diameter between 1.7 and 2 mm, a dedicated thinner 5-Fr sheath was used (Terumo Corporation, Tokyo, Japan). Sheath was inserted under ultrasound guidance according to the Seldinger technique.

When patient height was between 175 and 195 cm, “proximal” TRA (pTRA) was performed: radial artery puncture was performed 5 to 10 cm proximally to the usual radial puncture site at an extra-muscular location. When patient height exceeded 195 cm, TFA was chosen.

Following sheath insertion, an antispasmodic and antithrombotic mix of 1 mg of isosorbide dinitrate, 2.5 mg of verapamil, and 3000 IU of heparin was injected in the radial artery through the sheath, after dilution in 20 mL of blood. No additional heparin was injected during the procedure.

TR PAE was performed using a 125 or, when needed, a 135-cm long 5-Fr catheter (Merit medical), a hydrophilic angulated 0.035 guide wire (Terumo Corporation), a 150-cm long microcatheter (Merit medical), a 0.014′ micro guide wire (Boston Scientifics, Malborough, MA, USA), and 300–500 μm calibrated trisacryl microparticles (Merit Medical) until complete stasis, as previously described [13]. Coil protection was used in elective cases to prevent extra-prostatic non-target embolization [14,15].

TFA was performed on the right side, using a 5-Fr sheath (Terumo Corporation, Tokyo, Japan, or Cook Medical, Bloomington, IN, USA), a 100-cm long 5-Fr catheter (Terumo Corporation), and a 130-cm long microcatheter (Merit Medical).

Technical success for TRA was defined as completion of the procedure and at least unilateral prostatic artery embolization. Failure was defined as an incapacity for internal iliac or prostatic artery catheterization due to insufficient device length. In case of failure of TRA, the procedure was completed after conversion to TFA. 

After complete TR PAE, hemostasis was performed using a hemostatic band (TR Band^®^, Terumo Corporation, or Prelude Sync^®^, Merit Medical) as follows: initial inflation of 20 mL of air in the compressive valve was performed to permit a bleeding free sheath retrieval, followed by progressive deflation according to the “patent hemostasis protocol”, previously described [16]: when pulsatile reflux of blood was observed through the arteriotomy, 0.5 mL of air was re-inflated to stop the reflux, and palpation of distal radial pulse was reached to confirm artery patency. A first 5 mL deflation was performed at 30 min of compression and a final deflation of the remaining volume at 45 min. In case of bleeding at puncture site during deflation, 2 mL was re-inflated to stop the bleeding, and deflation was reinitiated 15 min later until complete hemostasis. At time to hemostasis, band was retrieved, puncture site cleaned, and a bandage was put on. Prior to this study, the deflation protocol recommended in both institutions for 5-Fr TR embolization procedures was of a 90 min duration, with increments in deflation of 3 mL and the remaining volume at 60, 75, and 90 min.

When patients showed arterial hypertension during hemostasis, no measure was taken to lower blood pressure. Control ultrasound before discharge was performed in selected cases, when radial/distal pulses were not palpated after hemostasis (suspicion of RAO) or when bleeding occurred during/following deflation, in order to rule out pseudo-aneurysm at the puncture site.

Patients were discharged after voiding > 200 mL, 30 to 45 min after hemostasis, and a form was provided to report any adverse event occurring after discharge.

Follow-up consult was performed at 1, 6, and 12 months to assess clinical improvement and monitor adverse events: severity was defined according to the Society of Interventional Radiology Clinical Practice Guidelines [17]. The same documentation as in pre-operative evaluation was obtained, and radial access site DUS monitored RAP and absence of pseudo-aneurysm at one month. Criteria for clinical success were IPSS score decrease of 8 points, QoL score decrease of at least 1 point or value ≤3, increase of 2.5 mL/s of Qmax, and successful retrieval of indwelling catheter 15 days after PAE or resolution of hematuria.

Agreement of the Institutional Review board was obtained for this study. 

The primary endpoint was safety of a 45 min deflation protocol for hemostasis, described as absence of major adverse events, such as acute hematoma or hand pain requiring hospitalization, and comparable rates of minor adverse events to what was previously described in literature. The secondary endpoint was the feasibility of PAE using TRA, consisting of technical success and no need for conversion to TFA.

### Statistical Analysis 

Logistic regression was used to determine predictors for access site adverse events. Univariate and multivariate analyses were performed using R software, version 4.1.1. Results are expressed as Odd Ratio (OR) value [95% Confidence Interval, IC] and their *p* value. A *p* value < 0.05 was considered significant. 

## 3. Results

Among the cohort of consecutive patients referred for PAE, TFA was chosen over TRA in 11/311 (3.6%) patients: one patient was 197 cm tall, one had a history of kinking of the abdominal aorta, one had a history of occlusion of the left subclavian artery, and the 8 remaining patients had advanced atheroma. Among the TRA cohort, technical success was achieved in 296/300 (98.7%) cases. Bilateral embolization was achieved in 294/296 (99.3%) cases. The four cases of failure of TRA included one case of a painful radial loop preventing completion of the procedure through TRA under local anesthesia, one case showing undocumented occlusion of the left subclavian artery preventing catheterization, one case of combined subclavian artery kinking and aortic aneurysm/tortuosity preventing catheterization of the descendant thoracic aorta, and one case where cannulation of the internal iliac artery was not achieved on one side because of significant iliac tortuosity making the 135-cm long 5-Fr catheter too short for selective angiography (patient’s height was 185 cm). Procedural characteristics of the cohort are presented in Table 2. 

**Figure 1:**
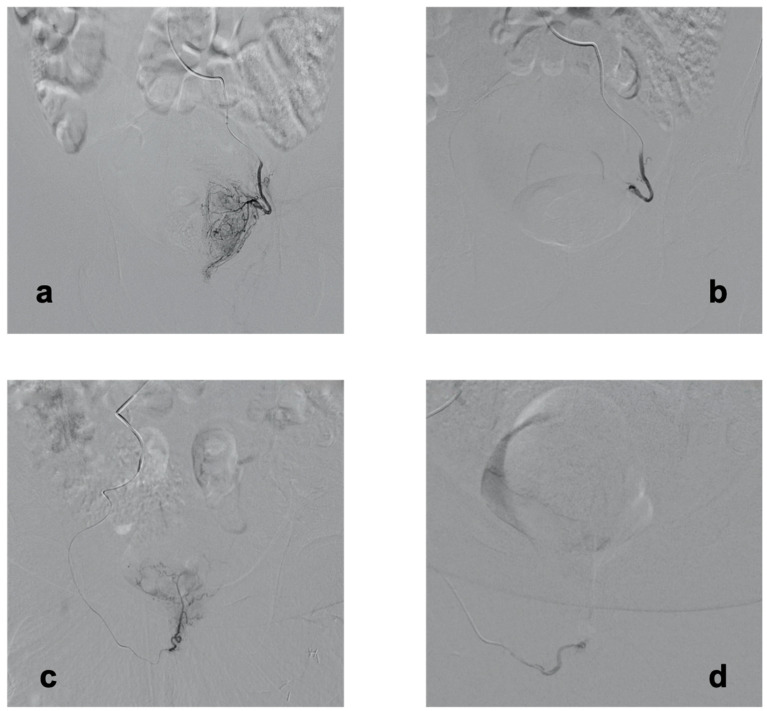
Selective prostatic artery angiograms during transradial prostatic artery embolization. All angiograms are performed on anteroposterior view. (**a**) Selective digital subtraction angiography of the left prostatic artery, showing a full uptake of the left hemi-prostate. (**b**) Selective digital subtraction angiography of the left prostatic artery following embolization, confirming complete stasis in the artery and disappearance of the uptake. (**c**) Selective digital subtraction angiography of an accessory right prostatic artery, arising from the distal part of the internal pudendal artery, and feeding both sides of the prostate. (**d**) Post-embolization selective digital subtraction angiography of the right accessory prostatic artery, confirming complete stasis and absence of uptake.

An angiography review revealed that 48/300 (16%) patient had an accessory prostatic artery originating from a distal branch of the internal pudendal artery (“distal accessory PA”) (Figure 1). No lack of microcatheter length was observed, and all but one were successfully catheterized and embolized.

The mean procedure time was 95 ± 26.1 (45–195) min, mean fluoroscopy time and dose-area product (DAP) were 35 ± 14.7 (11–97) min and 16,225 ± 12,126.3 (2959–81,608) μGy·m^2^. Mean time to discharge was 80 ± 6 (75–90) min. Clinical success at one month following TR PAE was 258/300 (86%).

### Adverse Events

There was no major adverse event. There was no case of stroke or any neurological event, including acute pain in the left hand. 

All radial pulses were palpated at time to hemostasis. DUS was performed in 2/300 patients prior to discharge because of bleeding during deflation. Mild hematoma at the puncture site was observed in 30 (10/%) cases, and all appeared the next day after discharge. Among them, 4/30 patients were under an anticoagulant and 3/30 patients were under aspirine; none were under clopidogrel medication. Univariate or multivariate logistic regression analysis did not find any significant predictor among age, height, radial artery diameter, pTRA, history of diabetes, arterial hypertension, smoking, dyslipidemia, anticoagulant, or antiaggregant medication for occurrence of hematoma. 

There were 2/300 (0.7%) cases of asymptomatic thrombosed pseudo-aneurysm (P-A) of the anterior wall of the radial artery at the puncture site, both diagnosed by DUS: one was observed at day 2 in a patient under VKA medication, who presented in clinic because of a mild hematoma occurring the day before. This P-A had disappeared at control DUS at one month. The second P-A was observed at one-month follow-up DUS in a patient who was under DOA medication.

There were three cases (1%) of arteritis of the left radial artery, manifested by mild swelling and pain in the left arm and wrist along the artery pathway. All three cases occurred after discharge, between day 3 and day 5 following PAE, and one was associated with RAO, which was diagnosed by DUS at the clinic at day 5. After infection was ruled out, they were treated by oral non-steroidal anti-inflammatory drugs, antibiotics, and painkillers and subcutaneous heparinotherapy for the case of associated thrombosis. All evolved favorably under medical treatment within 10 days, but RAO resolved only subcompletely because the patient decided to stop heparinotherapy at day 15. 

There were 10/300 (3.3%) cases of radial artery occlusion: nine were asymptomatic and monitored at one month by DUS, and the last one was the one associated with arteritis and diagnosed at day 5 by DUS. No medication was given at one month to treat the asymptomatic occlusions because the diagnosis was considered too late for initiating anticoagulant treatment and because occlusions were asymptomatic. Two RAOs persisted at the 6-month control DUS, one persisted at one-year, the seven other patients were lost to follow-up. Among them, the radial artery diameters varied between 1.85 and 3 mm. Univariate logistic regression analysis found that history of smoking, radial artery diameter < 2 mm (compared to diameter between 2 and 3 mm or <3 mm), and occurrence of hematoma were significant predictive factors for the occurrence of occlusion (OR = 5.63 CI [1.56; 22.62], *p* = 0.009; OR = 4.51 CI [0.92; 17.75], *p* = 0.04 and OR = 4.17 CI [0.86; 16], *p* = 0.046). Multivariate logistic regression found smoking to be a significant predictive factor for the occurrence of RAO (OR = 6.52 CI [1.49; 31.15], *p* = 0.013).

Access site and overall embolization-related adverse events are shown in Table 3 and Table 4. Post-embolization syndrome, including mild fever, fatigue, pelvic/anal pain, urethral burning, pollakiuria, and constipation occurring during the first 10 days were not considered adverse events.

## 4. Discussion

### 4.1. Transradial Access

This study confirmed feasibility and safety of TRA during PAE. There was a low rate of technical failure leading to the conversion to TFA (1.3%). Over the two studies available in the literature on TRA during PAE, Isaacson et al. [9] and Bhatia et al. [11] reported no conversion to TFA in 19 and 32 patients, respectively, but their cohorts were smaller. Still, Bhatia reported 2/32 (6%) conversions to transulnar access.

The results in this study are similar to those of two recent studies on TRA during IR procedures, reporting a 1/91 (1%) [18] and 4/749 (0.5%) [8] rate of conversion to TFA. In the present study, patients in whom TRA failed were 64, 76, 81, and 91 years old. This might suggest that risk for failure may increase with advanced age, but it needs to be confirmed by additional studies. A 1.3% rate of failure in TRA may be of debate, but it is to be balanced with the many benefits of this approach: in addition to those previously described [8], significantly lower DAP and faster ambulatory discharge may be observed compared to TFA [11].

Procedure characteristics were not compared to those of the 11 patients treated using TFA because this TFA cohort was too small for a comparison.

### 4.2. Adverse Events

This study showed that access site and overall adverse events following a 45 min time to hemostasis in TRA were all minor. To our knowledge, there is no recommended time to hemostasis for 5-Fr TRA in IR procedures. Even though the basic deflation protocol that was locally recommended for previous 5-Fr TR embolization procedures was of a 90 min duration, all TR PAE procedures were performed in both institutions of this study using this 45 min deflation protocol, and the results could therefore not be compared to those of PAE procedures using a 90 min deflation protocol. Isaacson et al. described a deflation protocol, but the total duration was not detailed [9]. Nakhaei et al. described in 91 TRA for uterine fibroid embolization (UFE) a 40 min deflation protocol, with deflation increments at 30, 35, and 40 min and with safe results [18], which supports our results.

Hematoma occurred in 10% of patients in this study. Isaacson et al. reported 11%, and Bhatia et al. 9.4% (TRA) and 12.5% (TFA) [9,11]. 

In patients manifesting hematoma, anticoagulant, aspirine, or clopidogrel medication were not predictive factors. Still, patients under DOA or VKA medications may be at increased risk of peudoaneurysm at the puncture site. These findings need to be confirmed in further studies.

To our knowledge, there is no report describing pTRA in the literature. pTRA is of benefit when the patient’s height is at least 175 cm, as it may prevent the lack of catheter length for cannulation of the IIA or distal accessory PA. Findings in this study suggest that the safety of this puncture seems acceptable. Further studies are needed on this topic.

There was a 3.3% rate of RAO. Isaacson et al. and Bhatia et al. reported none, but their cohorts were smaller (*n* = 19 and *n* = 32) [9,11]. Thakor et al. reported a 0.3% of RAO (*n* = 749) [8], using “patent hemostasis” [16] during TR embolization procedures. These previous results on RAP following TRA were based on post-procedural or follow-up clinical examination, which may underestimate the incidence of RAO compared to DUS, as collateral supply via the superficial palmar arcade may provide retrograde arterial flow in the radial artery at palpation site, distally to the occlusion and maintain distal pulse. This hypothesis may explain why all patients in this study, including those who encountered RAO, had a pulse palpated at time to discharge. Immediate RAO may not be excluded, and control DUS prior to discharge may be of interest to unmask this event. RAO could then be treated early by anticoagulants with a high chance of resorption of the thrombosis. The results in this study may suggest that part of RAO persist in time, when diagnosed “too late” at one month. Some studies in the literature reported the opposite: most cases spontaneously resolved between discharge, 24 h, 1-, and 3-months control DUS [18,19].

This finding on RAO indicates the need for adapted deflation protocols for TR PAE: shorter time to first increment in deflation and/or overall time of compression may be considered.

Alternative maneuvers to reduce RAO were previously described in 5–6F cardiology procedures in randomized studies, such as subcutaneous preprocedural injection at puncture site (*n* = 188) [20] or intra-arterial pre-hemostasis injection (*n* = 1706) [21] of 500 μg of nitroglycerin, ipsilateral ulnar artery compression adding to patent artery compression (*n* = 3000) [19], with significant reduced incidence of RAO (5.4 vs. 14.4%, 8.3 vs. 11.7%, and 0.9% vs. 3%). Heparin sheath injection may play a role [22] (5000 IU in most cardiology procedures vs. 3000 in our study). At last, additional IV injections of 1000 units of heparin every 30 to 60 min during the procedure, to reduced risk of clotting, may be considered.

History of smoking, small caliber artery, and occurrence of hematoma were found to be predictors for RAO. Elective control DUS prior to discharge or within the first days following the procedure for patients in these situations may be of interest in order to early diagnose asymptomatic RAO and start anticoagulant treatment to recover artery patency.

This study reported short-term clinical improvement of 86%. These results are comparable to data in the literature [4,5,9,11].

This study has its limitations, starting with its retrospective nature, its non-randomized nature, the limited follow-up period, and its patients lost to follow-up. This study lacked control groups: this cohort was not compared to another cohort of patients benefitting TRA using a 90 min deflation protocol or TFA. RAP was not monitored at one year in all patients encountering occlusion, which may overestimate the RAO.

## 5. Conclusions

The safety of transradial access during outpatient PAE using a 45 min time to hemostasis was confirmed and may help to shorten the time to discharge. When purposely chosen, TRA is feasible in most cases of PAE. A low incidence of radial artery occlusion was still observed and may be favored by smoking patients, small caliber arteries, and the occurrence of hematoma. These findings need confirmation by additional studies, and there is a need for comparison between techniques for hemostasis in randomized designs.

## Figures and Tables

**Table 1:** Baseline characteristics of study cohort prior to PAE.

Variable	Study Cohort (*n* = 300)
Age, years	68 ± 9.7 (47–102)
Height, cm	176 ± 6.3 (158–192)
Radial artery diameter at puncture site, mm	2.5 ± 0.3 (1.7–3.6)
Medication at procedure	53 (17.7)
Aspirin medication	22 (7.3)
Clopidogrel medication	1 (0.3)
Aspirin and clopidogrel medication	2 (0.7)
Aspirin and ticagrelor medication	2 (0.7)
DOA/VKA/Heparin	26 (8.7)
Indication for PAE	
Bothersome LUTS	241 (80.3)
Urinary retention	51 (17)
Macroscopic hematuria	8 (2.7)
	
IPSS	19 ± 6.9 (4–35)
QOL score	6 ± 1.1 (2–7)
IIEF-15	45 ±19.3 (4–77)
Prostate volume, mL	92 ± 45.2 (22–280)
Maximum urinary flow, mL/s	8 ± 5 (2.4–31)
Post-voiding residue, mL	98 ± 123 (0–810)
Total PSA, ng/mL	7 ±5.6 (0.31–28)

Note: values are presented as mean ± SD (range) or as number, *n* (%). PAE: prostatic artery embolization; DOA: direct oral anticoagulant; VKA: vitamine K ntagonist; LUTS: lower urinary tract symptoms; IPSS: international prostatic symptoms score; QOL: quality of Life; IIEF: international index of erectile function; PSA: prostatic specific antigen.

**Table 2:** Procedure characteristics of the study cohort.

Variable	Study Cohort (*n* = 300)
Technical success	296 (98.7)
Conversion to TFA	4 (1.3)
Proximal TRA	149 (49.7)
Distal accessory PA	48 (16)
	
Procedure time, min	95 ± 26.1 (45–195)
Fluoroscopy time, min	35 ± 14.7 (11–97)
DAP, μGy·m^2^	16,225 ± 12,126.3 (2959–81,608)
Radiation skin entry, mGy	1557 ± 1098.6 (238–5958)
	
Closure device	
TR Band	199 (66.3)
Prelude sync	101 (33.7)
Mean time to discharge after completion of procedure, min	80 ± 6 (75–90)

Note: values are presented as mean ± SD (range) or as number, *n* (%). DAP: dose-area product; min: minute; Gy: Gray; PA: prostatic artery.

**Table 3:** Access site adverse events.

Variable	Cohort (*n* = 300)
Stroke	0
Hand pain	0
Hematoma after discharge	30 (10)
Thrombosed pseudo-aneurism at puncture site	2 (0.7)
Arteritis	3 (1)
Radial artery occlusion	10 (3.3)

Note: values are represented as mean ± SD (range) or as number, *n* (%).

**Table 4:** Embolization-related adverse events.

Variable	Cohort (*n* = 300)
Acute urinary retention	0
Urinary tract infection	2 (0.7)
Hematuria	5 (1.7)
Bladder ischemia	0
Rectorrhagia	0
Rectal ischemia	0
Balanitis	2 (0.7)
Penile glans necrotic ulcer	0
Erectile dysfunction	0
Hematospermia	6 (2)
Anejaculation	0

Note: values are presented as mean ± SD (range) or as number, *n* (%).

## Data Availability

Data containing patient characteristics prior and after the intervention, in addition to procedure characteristics, are available and can be found in the PACS and RIS of Hospital privé Geoffroy Saint-Hilaire and Hospital Saint-Louis, where the interventions occurred.

## Abbreviations

BPH	Benign Prostatic Hyperplasia
PA	Prostatic Artery
PAE	Prostatic Artery Embolization
LUTS	Lower Urinary Tract Symptoms
AUR	Acute Urinary Retention
IR	Interventional Radiologist
IRB	Institutional Review Board
DSA	Digital Subtracted Angiography
AP view	Antero-posterior view
CBCT	Cone Beam Computed Tomodensitometry
IIA	Internal Iliac Artery
TR	Transradial
TRA	Transradial Access
TFA	Transrfemoral Access
pTRA	Proximal Transradial Access
RAO	Radial Artery Occlusion
DOA	Direct Oral Anticoagulant
VKA	Vitamine K Antagonist
DUS	Doppler Ultrasound
RAP	Radial Artery Patency
APA	Accessory Pudendal Artery
IPA	Internal Pudendal Artery
